# Extraction of dietary fibers from cassava pulp and cassava distiller’s dried grains and assessment of their components using Fourier transform infrared spectroscopy to determine their further use as a functional feed in animal diets

**DOI:** 10.5713/ab.21.0430

**Published:** 2022-01-05

**Authors:** Supattra Okrathok, Kanjana Thumanu, Chayanan Pukkung, Wittawat Molee, Sutisa Khempaka

**Affiliations:** 1 School of Animal Technology and Innovation, Institute of Agricultural Technology, Suranaree University of Technology, Nakhon Ratchasima 30000, Thailand; 2 Synchrotron Light Research Institute (Public Organization), Nakhon Ratchasima, 30000, Thailand

**Keywords:** Cassava Distiller’s Dried Grains, Cassava Pulp, Dietary Fiber, Fourier Transform Infrared Spectroscopy

## Abstract

**Objective:**

The present study was to investigate the extraction conditions of dietary fiber from dried cassava pulp (DCP) and cassava distiller’s dried grains (CDG) under different NaOH concentrations, and the Fourier transform infrared (FTIR) was used to determine the dietary fiber components.

**Methods:**

The dried samples (DCP and CDG) were treated with various concentrations of NaOH at levels of 2%, 4%, 6%, and 8% using a completely randomized design with 4 replications of each. After extraction, the residual DCP and CDG dietary fiber were dried in a hot air oven at 55°C to 60°C. Finally, the oven dried extracted dietary fiber was powdered to a particle size of 1 mm. Both extracted dietary fibers were analyzed for their chemical composition and determined by FTIR.

**Results:**

The DCP and CDG treated with NaOH linearly or quadratically or cubically (p< 0.05) increased the total dietary fiber (TDF) and insoluble fiber (IDF). The optimal conditions for extracting dietary fiber from DCP and CDG were under treatment with 6% and 4% NaOH, respectively, as these conditions yielded the highest TDF and IDF contents. These results were associated with the FTIR spectra integration for a semi-quantitative analysis, which obtained the highest cellulose content in dietary fiber extracted from DCP and CDG with 6% and 4% NaOH solution, respectively. The principal component analysis illustrated clear separation of spectral distribution in cassava pulp extracted dietary fiber (DFCP) and cassava distiller’s dried grains extracted dietary fiber (DFCDG) when treated with 6% and 4% NaOH, respectively.

**Conclusion:**

The optimal conditions for the extraction of dietary fiber from DCP and CDG were treatment with 6% and 4% NaOH solution, respectively. In addition, FTIR spectroscopy proved itself to be a powerful tool for fiber identification.

## INTRODUCTION

Dietary fiber is defined as non-starch polysaccharides (NSPs) that cannot be digested by enzymes in the gastrointestinal tract of poultry. These polysaccharides can be found naturally in feedstuffs include cellulose, non-cellulosic polysaccharides such as hemicellulose, pectic substances, gums, mucilage, and a non-carbohydrate component lignin [1,2]. Dietary fiber is classified into water soluble dietary fiber (SDF) and insoluble dietary fiber (IDF), the content and composition of SDF and IDF vary with feedstuff type. Dietary fiber plays an important role in poultry physiology. Its effects depend on structures and physicochemical properties. In particular, IDF has been shown to improve gut health, litter and nutrient utilization, by increasing crop and gizzard activity, stimulating digestive enzyme production and enhancing bacterial fermentation in the hind gut [3]. Additionally, dietary fiber can improve the intestinal microbial balance when the fibers are fermented by resident anaerobic microflora and produce short chain fatty acids. These end products can lower the intestinal pH and in the long term would lead to a reduction in the population of pathogenic bacteria. Such effects promote the gut health and function positively on improving growth performance, including mitigate NH_3_ emission in excreta of poultry [4]. Therefore, alternative dietary fiber sources derived from agro-industrial by-products could be a useful feed to improve animal health and provide the benefits of good waste management.

Cassava by-products in the form of dried cassava pulp (DCP) and cassava distiller’s dried grains (CDG) are annually generated in large amounts (approximately 2 to 3 and 0.74 to 0.95 million tons per year) from starch factories and by the bioethanol production process in Thailand, respectively. These by-products contain high NSPs which are mainly composed of cellulose, hemicellulose, and lignin [5,6]. The fiber extracted from DCP and CDG can be further used in poultry diets as it provides health benefits and productive performance as well as good waste management. Previous studies reported several methods of extracting dietary fiber, unfortunately, there is little information available on the extraction of cassava by-products. A combination of enzymatic and solvent methods is usually used for dietary fiber extraction. For example, starch and protein are first removed by amylase and protease or a suitable solvent (such as a neutral, alkaline, acidic detergent). The different fiber extraction conditions affect the properties of dietary fiber in both composition and structure [7]. Daou and Zhang [8] reported that the extraction of dietary fiber from defatted rice bran and the alkaline pretreatment with NaOH were the factors amongst others (e.g. concentration and soaking time) which significantly affected the purity of fiber fractions. Samanta et al [9] stated that the alkaline extraction (NaOH solution) can yield xylan in agricultural by-products. Crude fiber assay is still being used today as the legal measure of fiber in grains and finished feeds of non-ruminant animals. However, it is not a good indicator because some parts of the fiber can ferment in the large intestine or caecum. Thus, the true fiber contents have attracted more interest because of the potential improvements in the accuracy of future measurements [1]. A power tool to identify the components in fiber could offer a rapid and reliable alternative technique.

Fourier transform infrared (FTIR) Spectroscopy has be come an attractive alternative to traditional methods since it is a rapid analytical technique which uses non-destructive samples and minimizes hazardous chemical use. The FTIR can provide information regarding the functional groups, chemical bonds, composition, structure, and quality of a product. Infrared (IR) spectra combined with chemometric techniques, such as principal component analysis (PCA), can be used to obtain accurate dietary fiber components [10–12]. FTIR Spectroscopy has been used to study the relationship of feed intrinsic structures pertaining to protein molecular structures, carbohydrates, and starch matrices [10], quantitative analysis of tapioca starch [13], and polysaccharide food additives [14]. It can be used to analyze the chemical composition of cell walls, the structure of natural fiber and fiber composition [15]. The application of FTIR to identify the fractions of dietary fiber from DCP and CDG would be a useful tool for the assessment of dietary fiber components.

The present study primarily focused on the investigation of the extraction conditions of dietary fiber from DCP and CDG by using different NaOH concentrations, with the purpose of producing dietary fiber. To the best of our knowledge, this is the only study of dietary fiber estimation using FTIR analysis to determine dietary fiber components combined with multivariate data analysis using PCA.

## MATERIALS AND METHODS

### Sample preparation

Fresh cassava pulp was obtained from the Korat Flour Industry Co., Ltd, Nakhon Ratchasima, Thailand. Fresh CDGs were obtained from the Thai ethanol power Pub Co., Ltd, Khon Kaen, Thailand. These were dried in a hot air oven at 55°C to 60°C for 2 d and then were ground to pass through a 1.0 mm mesh sieve before being stored at 4°C until further use. Prior to extraction, DCP and CDG were analyzed for dry matter, crude protein, ash, and ether extract according to the standard methods of AOAC [16]. The contents of total soluble and IDF were determined using the total dietary fiber Kit (K-TDFR-100A, Megazyme International Ltd., Wicklow, Ireland). Briefly, the samples (1 g) were treated with thermostable α-amylase and then incubated at 60°C with protease and amyloglucosidase to eliminate starch and protein components. The IDF was obtained by filtration and the residue was washed with warm distilled water. The SDF was precipitated with 95% ethanol and filtered. Total dietary fiber (TDF) was calculated as the sum of SDF and IDF. The chemical compositions of DCP and CDG are shown in Table 1.

### Experimental design and dietary fiber extraction

Dietary fiber from DCP and CDG were treated with various concentrations of NaOH at levels of 2%, 4%, 6%, and 8% using a completely randomized design with 4 replications of each. The extraction procedure was slightly modified from Daou and Zhang [8]. A dried sample (1.0 g) was pretreated with 5.0 mL NaOH solution at different concentrations (2%, 4%, 6%, and 8%), the mixture was soaked for 1 h at room temperature, then centrifuged, and the residue was washed to pH 7.0 with distilled water. The sample was suspended in phosphate buffer (pH 6.0) ratio 1:30, and α-amylase (EC 3.2.1.1, Megazyme, Ireland) was added, then the mixture was incubated at 95°C in a boiling water bath for 1 h. The sample was allowed to cool at room temperature. The dietary fiber was precipitated in 95% ethanol at 60°C for 1 h, then was cooled to room temperature, and centrifuged. After centrifugation, the residue was washed with 78% ethanol, 95% ethanol and acetone, respectively, and finally dried at 55°C to 60°C overnight.

### Analysis of dietary fiber content

The DFCP and the DFCDG were analyzed to determine the amounts of TDF, SDF, and IDF by using a total dietary fiber Kit (K-TDFR-100A, Megazyme International Ltd., Ireland), according to the manufacturer’s instructions.

### Fourier transform infrared spectroscopy

The infrared spectra were collected using attenuated total reflectance (ATR)-FTIR spectroscopy with single reflection ATR sampling module and coupled with a DTGS detector over the measurement range from 4,000 to 400 cm^−1^. The measurements were performed with a spectral resolution of 4 cm^−1^ with 64 scans co-added (Bruker Optics Ltd, Ettlingen, Germany). OPUS software was used for data acquisition and the spectra evaluation. The spectral changes of the functional groups were performed at the integral area of each peak such as cellulose, hemicellulose, lignin, and starch by using OPUS software.

### Principal component analysis

The FTIR spectra were exported to the Unscrambler X 10.5 (CAMO, Oslo, Norway) for using PCA analysis. The spectral data were preprocessed by taking the 2nd derivative with Savitzky-Golay method (3rd polynomial, 9 smoothing points), normalization with extended multiplicative signal correction and PCA were performed for the determination of a significant variation between the spectra sets. In this study, PCA was used to compare the FTIR spectra of dietary fiber sources under treatment with different conditions of NaOH solution (2%, 4%, 6%, and 8%). The output of PCA can be presented as the sources of variability of data which were concentrated into the principal component (PC). The spectra were processed using the second derivative and vector normalized by the Savitzky–Golay method, and using the third polynomial and nine smoothing points setting [17].

### Statistical analysis

The experimental design was completely randomized design. Determinations for analysis were made using a total of 4 replicates. Statistical analyses of dietary fiber contents (TDF, SDF, and IDF) and peak area proportions from integrating FTIR spectra were performed using SPSS software version 18.0 [18]. Data were analyzed using one-way analysis of variance followed by Tukey’s tests. Orthogonal polynomials were also used to assess linear, quadratic, and cubic effects of NaOH levels. Values were statistically different at p<0.05.

## RESULTS AND DISCUSSION

### The components of dietary fiber extracted from dried cassava pulp and cassava distiller’s dried grains

The DCP and CDG were treated with different concentrations of NaOH solution (2%, 4%, 6%, and 8%) to determine the optimal conditions for dietary fiber extraction. The contents of TDF, SDF, and IDF after extraction are shown in Table 2. The DFCP consisted of an increase in TDF (cubic, p = 0.032) and IDF (linear, p = 0.001) in response to increased NaOH levels. It revealed that DFCP derived from treated DCP with NaOH at concentrations of 6% and 8% produced significantly greater amounts of TDF and IDF than 2% NaOH (p<0.05). In addition, the DFCDG also comprised an increase in TDF (cubic, p = 0.009) and IDF (quadratic, p<0.001). The DFCDG obtained by treatment CDG with 4%, 6%, and 8% NaOH produced higher contents of TDF and IDF than 2% NaOH (p<0.05). However, the SDF content in both DFCP and DFCDG showed no significant differences (p>0.05) between treatments.

This study showed that the optimum levels of NaOH for the extraction of dietary fiber from DCP and CDG were 6% to 8% and 4% to 8%, respectively. The IDF represented a major component in both dietary fiber sources. In general, some hemicellulose such as β-glucans, pectin, and gums are defined as SDF, whilst cellulose and lignin are defined as IDF [1,19]. Cellulose is the main structural constituent in plant cell walls, and also in cassava by-products [1,5,6]. Previous studies have shown that defatted rice bran treated with 0.6% NaOH solution can produce the maximum yield and purity of TDF, SDF, and IDF [8]. Samanta et al [9] demonstrate that the extraction agricultural by-products with NaOH solution resulted in recovery of more than 90% of original xylan in plant materials. The results were similar to the highest xylan recovery from sugarcane bagasse [20]. Harun and Geok [21] stated that rice straw treated with NaOH obtained the highest glucan and lower lignin composition.

### FTIR Spectra of dietary fiber extracted from dried cassava pulp and cassava distiller’s dried grains

**Figure 1:**
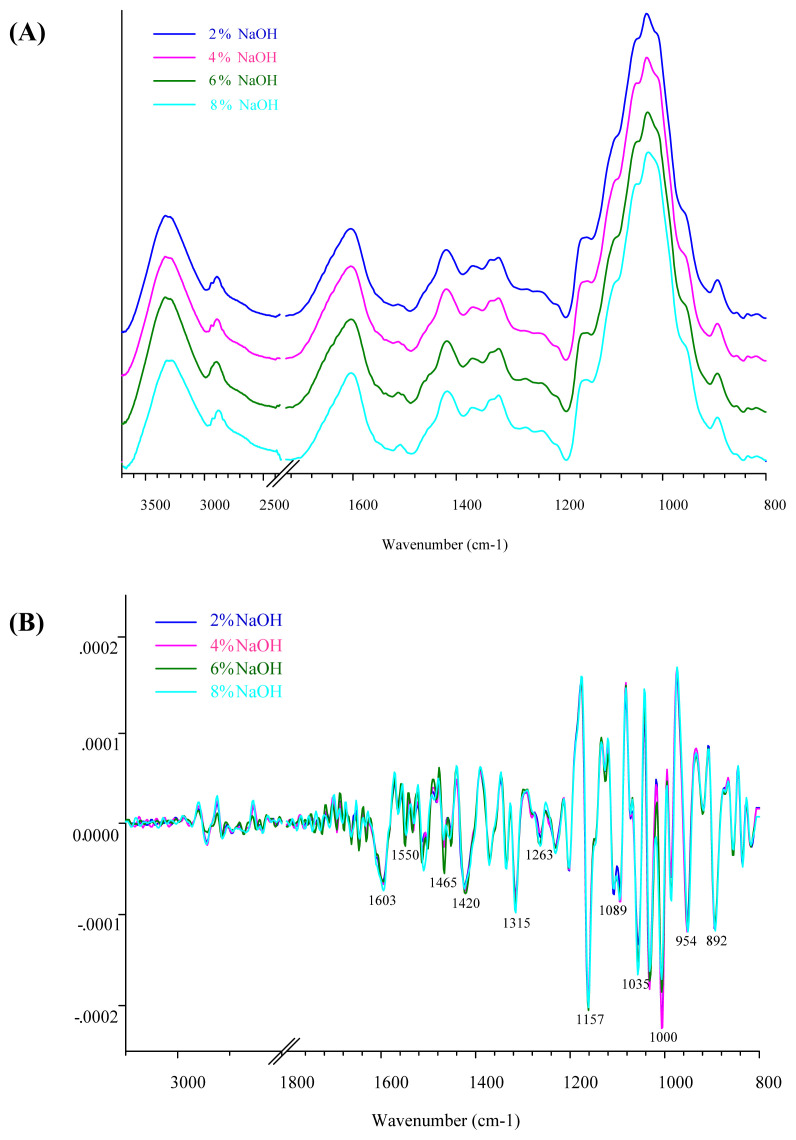
Fourier transform infrared (FTIR) spectra of cassava pulp extracted dietary fiber (DFCP) treated with NaOH solution at concentrations 2%, 4%, 6%, and 8%, (A) Average original FTIR spectra of DFCP, (B) The 2nd derivative spectra of DFCP. The infrared (IR) spectra detected in spectra region from 4,000 to 400 cm^−1^, resolution 4 cm^−1^, with 64 scans.

**Figure 2:**
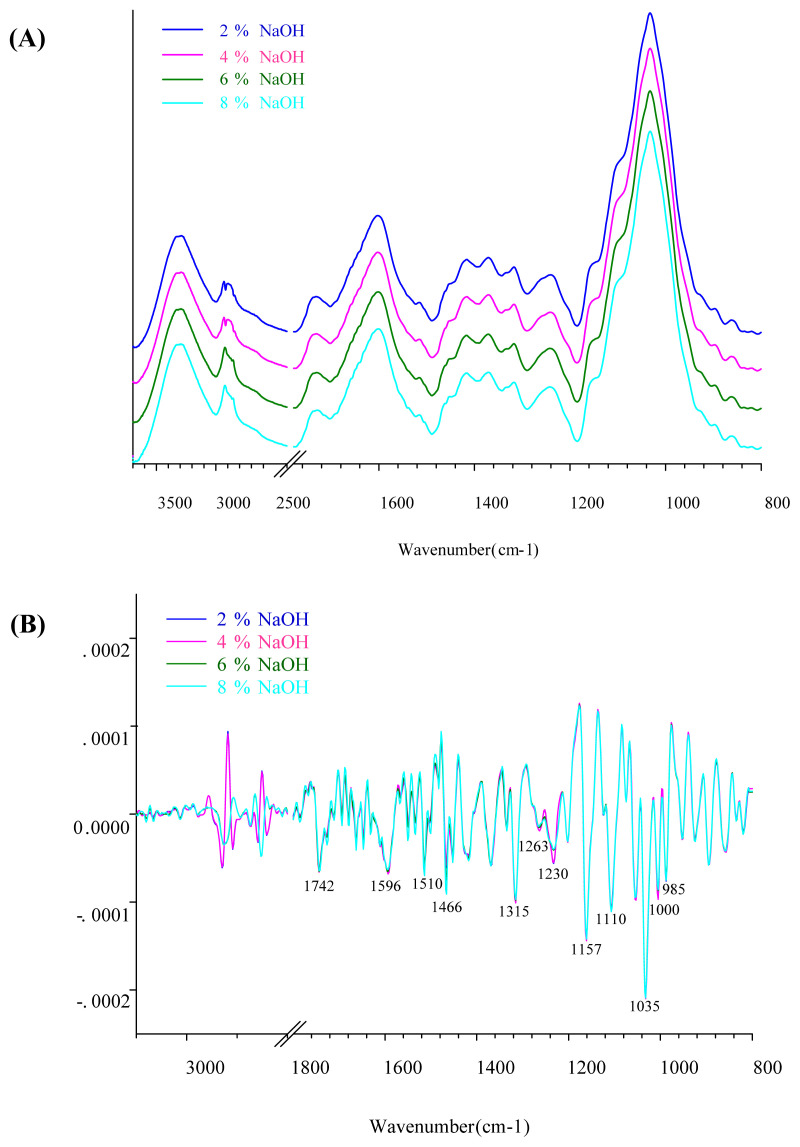
Fourier transform infrared (FTIR) spectra of cassava distiller’s dried grains extracted dietary fiber (DFCDG) treated with NaOH solution at concentrations 2%, 4%, 6%, and 8%, (A) Average original FTIR spectra of DFCDG, (B) The 2nd derivative spectra of DFCDG. The IR spectra detected in spectra region from 4,000 to 400 cm^−1^, resolution 4 cm^−1^, with 64 scans.

The band assignment of FTIR spectra of isolated dietary fiber is shown in Table 3. The spectral features of DFCP and DFCDG are shown in Figures 1 and 2. In this study, FTIR spectra were used to detect the extracted dietary fiber treated with NaOH solution in the spectral region of 4,000 to 400 cm^−1^. A total of 428 and 434 FTIR spectra of DFCP and DFCDG were analyzed. The fingerprint regions of specific interest in this study were between 1,700 and 800 cm^−1^, although many absorption bands associated with various NaOH solutions from vibrational modes in the wavelength region are also present in DFCP and DFCDG. The FTIR spectra measurements were carried out to reveal the molecular characteristics of functional groups of dietary fiber such as cellulose, hemicellulose and lignin. This technique can give information about the functional groups of C–O, C–O–C glycoside, and C–C from cellulose, hemicellulose, lignin, starch, and glucose in extracted dietary fiber [22–24].

The semi-quantitative analysis of FTIR spectra was car ried out by using OPUS software. The presented FTIR spectra were used to average the single spectra of each sample. The wavenumber of the FTIR spectra was determined to be in the region of 3,500 to 800 cm^−1^ for the peak area integration, and the total area of integrated peaks was defined as 100%. The peak area units were expressed as relative proportions of the components in DFCP and DFCDG. The results of the semi-quantitative analysis of DFCP and DFCDG using FTIR spectra in term of proportions (%) of the functional groups are presented in Table 4. The results showed that DFCP comprised of an increase in C–H bending of crystalline versus amorphous structure of cellulose (cubic, p<0.001), C–O stretching of hemicellulose (cubic, p = 0.001) and C–O–C glycoside, C–O and C–C stretching of cellulose (cubic, p = 0.029) in response to increased NaOH levels. The DFCP derived from treated DCP with 6% NaOH yielded significant proportions of C-H bending of crystalline cellulose and C–O–C glycoside, C–O and C–C stretching of cellulose compared to 2% and 4% NaOH (p<0.05), but there was no significant difference when compared to 8% NaOH treatment. While the proportions of the components in DFCDG with peak area integration revealed an increase in C–H stretching (linear, p = 0.002), C–H bending of crystalline cellulose (linear, p = 0.015), C–O–C glycoside, C–O and C–C stretching of cellulose (cubic, p = 0.033), C–O stretching of starch (linear, p<0.001) and vibration of the pyranose ring (quadratic, p = 0.004). The DFCDG derived from treated CDG with 4% NaOH resulted in a significant yield of C–O–C glycoside, C–O and C–C stretching of cellulose compared to 2% NaOH (p<0.05). However, no significant differences were observed in crystalline structure of cellulose in response to the concentration of 2% and 4% NaOH. These results were similar to the chemical composition of DCP treated with 6% NaOH and CDG treated with 4% NaOH, which showed higher functional groups of cellulose. It has been well-established previously that IDF commonly includes cellulose [7].

### PCA analysis and extracted dietary fiber components

In this study, the DCP and CDG treated with NaOH solutions at a concentration of 2%, 4%, 6%, and 8% were identified by their spectral distribution by using PCA. The PCA scores were plotted to characterize the sample relationships between the spectra and the dietary fiber extraction treatments. The FTIR characterizes chemical structure by identifying the functional groups present in each sample.

**Figure 3:**
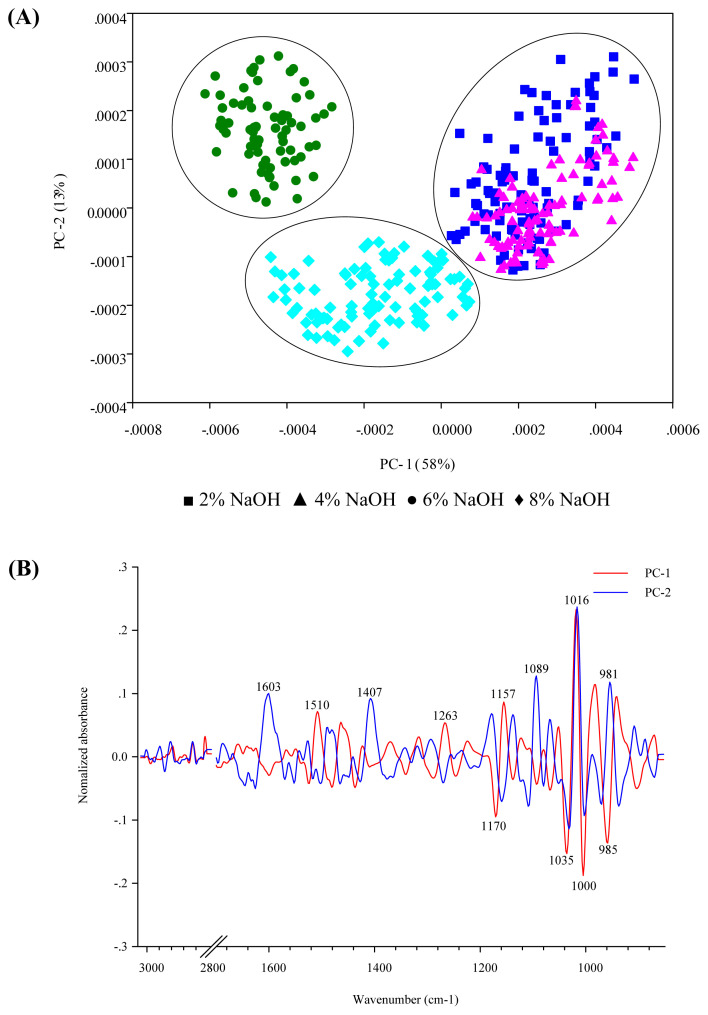
Principal component analysis (PCA) scores scatter plot of Fourier transform infrared (FTIR) spectra of cassava pulp extracted dietary fiber (DFCP) treated with NaOH solution at concentrations 2%, 4%, 6%, and 8%, (A) PCA scores plot, (B) PCA loading plot.

The results of the PCA scores from DFCP are presented in Figures 3. The variability of PC-1 and PC-2 accounts for 58% and 13%, respectively. The scores plot of DFCP derived from DCP treated with 6% and 8% NaOH appear clearly in the negative PC-1 score plot, while the scores plot of 2% and 4% NaOH treatments are clearly separated in the positive PC-1 score plot. The highest negative loading plot in PC-1 was observed in the C–O–C stretch of cellulose (centered at 1,170 cm^−1^), C–O vibrations of cellulose (centered at 1,035 cm^−1^), C–O and ring stretching (centered at 1,000 and 985 cm^−1^), which was oppositely correlated with the positive score plots in DFCP treated with 2% and 4% NaOH group from the second derivative spectrum. While the treatment using 6% NaOH shows the scores plot differ from 8% NaOH and almost appear on the positive side of PC-2. The positive loading plot in PC-2 reveals O–H bending of adsorbed water (centered at 1,603 cm^−1^), C–H bending of crystalline cellulose (centered at 1,407 cm^−1^), C–O and C–O–C stretching (centered at 1,089 and 1,016 cm^−1^), and C–O stretching of starch (centered at 981 cm^−1^).

**Figure 4:**
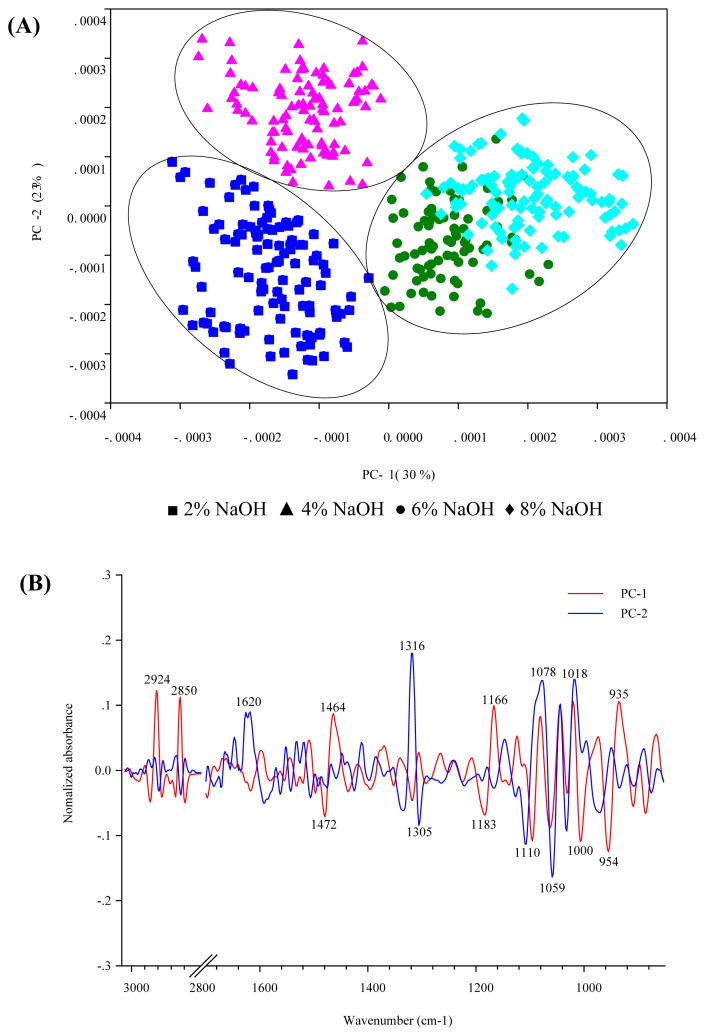
Principal component analysis (PCA) scores scatter plot of the Fourier transform infrared (FTIR) spectra of cassava distiller’s dried grains extracted dietary fiber (DFCDG) treated with NaOH solution at concentrations 2%, 4%, 6%, and 8%, (A) PCA scores plot, (B) PCA loading plot.

The score plot of the FTIR spectra of DFCDG is presented in Figures 4. The variation of spectra in PC-1 and PC-2 accounts for 30% and 23%, respectively. The scores plot of DFCDG derived from CDG treated with 2% and 4% NaOH appear in the negative PC-1 score plot, while the scores plot of 6% and 8% NaOH appears separately in the positive PC-1 score plot. The negative loading plot in PC-1 reveals C–H bending (centered at 1,472 cm^−1^), C–O and ring stretching (centered at 1,000 cm^−1^), and vibrations of the pyranose ring, glucose ring stretch (centered at 954 cm^−1^). The DFCDG derived from CDG treated with 4% NaOH show the scores plot differs from that of 2% NaOH and almost appears in the positive PC-2 score plot. The positive loading plot in PC-2 reveals O–H bending of adsorbed water (centered at 1,620 cm^−1^), C–H bending of crystalline cellulose (centered at 1,316 cm^−1^), and C–O stretching and C–C stretching of cellulose (centered at 1,078 and 1,018 cm^−1^).

The results of DCP and CDG using different NaOH solu tions indicate that PCA analysis of FTIR spectroscopy reveals differences in DCP and CDG treated with 6% and CDG 4% NaOH solutions, respectively. These results are related to the semi-quantitative analysis by integral area obtained from the spectra. The results also show the main components of cellulose in both dietary fiber sources. These results are consistent with those of Uthumporn et al [25], who found the predominant content of NSPs extracted from sago palm flour were cellulose, hemicellulose, pectin, and lignin by using FTIR. Chirinang et al [26] reported that the FTIR spectrum of dietary fiber from cassava pulp appears in the band of 1,031 to 1,005 cm^−1^. This band is the fingerprint of polysaccharides. These results show that FTIR spectroscopy can be used as a very reliable and quick tool for evaluating and monitoring dietary fiber.

## CONCLUSION

It was demonstrated that DCP and CDG treated with 6% and 4% NaOH solutions respectively, obtained the highest TDF and IDF contents. These results are associated with the FTIR spectra integration from a semi-quantitative analysis, which indicates the spectral distribution of dietary fiber components of DCP treated with 6% NaOH and CDG treated with 4% NaOH have a clearly separated spectral distribution. This study reveals that FTIR spectroscopy is a useful and rapid technique for fiber identification and the semi-quantitative analysis.

## Figures and Tables

**Table 1:** The chemical compositions of dried cassava by-products

Compositions (%)	Cassava pulp	Cassava distiller’s dried grains
Dry matter	93.79	92.26
Crude protein	2.30	10.14
Ash	1.87	16.58
Ether extract	0.22	1.02
Total dietary fiber	17.36	21.47
Soluble dietary fiber	2.29	3.09
Insoluble dietary fiber	15.07	18.38

**Table 2:** The contents of total, soluble and insoluble dietary fiber from cassava pulp extracted dietary fiber and cassava distiller’s dried grains extracted dietary fiber

Item	Concentration of NaOH levels				SEM	p-values			
	
	2%	4%	6%	8%		ANOVA	Linear^1)^	Quadratic^1)^	Cubic^1)^
Cassava pulp extracted dietary fiber									
Total dietary fiber	23.60^b^	24.29^b^	27.93^a^	27.60^a^	0.57	0.001	0.001	0.285	0.032
Soluble dietary fiber	3.65	2.57	3.19	2.84	0.19	0.501	0.501	0.188	0.365
Insoluble dietary fiber	19.95^b^	21.72^ab^	24.73^a^	24.76^a^	0.63	0.001	0.001	0.117	0.089
Cassava distiller’s dried grains extracted dietary fiber									
Total dietary fiber	22.85^b^	25.12^a^	25.33^a^	25.35^a^	0.30	<0.001	<0.001	<0.001	0.009
Soluble dietary fiber	2.38	2.59	2.33	2.32	0.06	0.313	0.368	0.356	0.152
Insoluble dietary fiber	20.46^b^	22.53^a^	23.00^a^	23.03^a^	0.31	<0.001	<0.001	<0.001	0.186

SEM, standard error of the means; ANOVA, analysis of variance.

1) Orthogonal polynomials were used to evaluate linear, quadratic, and cubic responses to the concentration of NaOH levels.

a,b Means with different superscripts in a row are significantly different (p<0.05).

**Table 3:** Infrared assignment of the main bands in Fourier transform infrared spectra

Wavenumber (cm^−1^)	Assignments	Reference
2,800 – 3,000	C–H stretching (aliphatic compounds)	Lammers et al [22] Abidi et al [27] Pouzet et al [12]
1,600 – 1,680	O–H bending of adsorbed water	Abidi et al [27] Pouzet et al [12]
1,514, 1,595	C–H deformation of lignin	Lammers et al [22]
1,300 – 1,500	C–H bending (crystalline versus amorphous structure of cellulose)	Abidi et al [27] Ying et al [28]
1,200 – 1,260	C–O stretching of hemicellulose	Corredor et al [23] Abidi et al [27] Cheikh Rouhou et al [29]
1,035 – 1,160	C–O–C glycoside in cellulose, C–O vibration of crystalline cellulose, C–O stretching and C–C stretching of cellulose	Corredor et al [23]
		Abidi et al [27] Ying et al [28] Cheikh Rouhou et al [29]
980 – 1,000	C–O and ring stretching modes, C–O stretching of starch	Lammers et al [22] Abidi et al [27]
800 – 960	Vibration of the pyranose ring, glucose ring stretch	Lammers et al [22] Corredor et al [23]

**Table 4:** The integral area from Fourier transform infrared spectra of extracted dietary fiber

Item	Concentration of NaOH levels				SEM	p-values			
	
	2%	4%	6%	8%		ANOVA	Linear^1)^	Quadratic^1)^	Cubic^1)^
Cassava pulp extracted dietary fiber									
C–H stretching	5.33	6.22	4.52	5.08	0.38	0.486	0.486	0.832	0.180
O–H bending of adsorbed water	8.86	8.87	7.79	8.52	0.17	0.065	0.116	0.237	0.052
C–H deformation of lignin	0.66	0.79	0.75	0.73	0.04	0.612	0.595	0.272	0.574
C–H bending of crystalline cellulose	18.20^bc^	17.06^c^	19.54^a^	18.57^ab^	0.27	0.001	0.018	0.769	<0.001
C–O stretching of hemicellulose	5.40^a^	5.04^b^	5.41^a^	5.32^a^	0.05	0.006	0.686	0.046	0.001
C–O–C glycoside, C–O and C–C stretching of cellulose	25.28^b^	25.51^b^	26.56^a^	25.69^ab^	0.17	0.015	0.060	0.046	0.029
C–O stretching of starch	17.96	18.21	18.75	19.11	0.24	0.346	0.082	0.905	0.831
Vibration of the pyranose ring	18.15	17.97	16.99	17.15	0.23	0.178	0.055	0.679	0.318
Cassava distiller’s dried grains extracted dietary fiber									
C–H stretching	3.56^ab^	3.89^a^	2.43^bc^	2.10^c^	0.25	0.007	0.002	0.345	0.087
O–H bending of adsorbed water	7.52	6.84	6.98	6.41	0.18	0.134	0.040	0.878	0.315
C–H deformation of lignin	1.03	0.68	0.81	0.87	0.08	0.528	0.648	0.252	0.506
C–H bending of crystalline cellulose	29.03^a^	27.30^ab^	27.71^ab^	26.38^b^	0.37	0.049	0.015	0.746	0.189
C–O stretching of hemicellulose	5.60	6.02	5.80	5.34	0.12	0.222	0.330	0.081	0.707
C–O–C glycoside, C–O and C–C stretching of cellulose	20.57^b^	22.58^a^	21.95^a^	22.28^a^	0.26	0.004	0.008	0.024	0.033
C–O stretching of starch	22.67^c^	23.23^bc^	24.36^ab^	25.64^a^	0.35	0.001	<0.001	0.356	0.807
Vibration of the pyranose ring	10.03^b^	9.44^b^	9.94^b^	10.98^a^	0.18	0.003	0.005	0.004	0.593

SEM, standard error of the means; ANOVA, analysis of variance.

1) Orthogonal polynomials were used to evaluate linear, quadratic, and cubic responses to the concentration of NaOH levels.

a–c Means with different superscripts in a row are significantly different (p<0.05).
